# Understanding the Dynamics of More Restrictive Medicines Policy: A Case Study of Codeine Up-Scheduling in Australia

**DOI:** 10.34172/ijhpm.2022.6872

**Published:** 2022-12-19

**Authors:** Kellia Chiu, Anne Marie Thow, Lisa Bero

**Affiliations:** ^1^School of Pharmacy, Faculty of Medicine and Health & Charles Perkins Centre, The University of Sydney, Sydney, NSW, Australia.; ^2^Menzies Centre for Health Policy, School of Public Health, Faculty of Medicine and Health & Charles Perkins Centre, The University of Sydney, Sydney, NSW, Australia.; ^3^Center for Bioethics and Humanities, University of Colorado Anschutz Medical Campus, Aurora, CO, USA.

**Keywords:** Codeine, Opioids, Scheduling, Drug Policy, Advocacy Coalition Framework, Australia

## Abstract

**Background:** There has been increasing concern over opioid-related harms across the world. In Australia in 2018, codeine-containing products were up-scheduled from over-the-counter access at pharmacies, to requiring a prescription. The drug regulator’s decision to up-schedule was contentious and widely debated, due to the potentially large impact on consumers and healthcare professionals. This study aimed to analyse influences on the codeine up-scheduling policy.

**Methods:** This retrospective policy analysis used the Advocacy Coalition Framework (ACF) to understand how policy actors with shared beliefs formed adversarial coalitions to shape policy. Data were drawn from documents (regulator policy documents, public submissions, news reports, organisational media releases and position statements) and semi-structured interviews with 15 key policy actors. Codes were generated relating to policy processes and actor beliefs; broad themes included the role of health professionals, perceptions of opioids, impact on consumers, and the role of government in healthcare.

**Results:** Two coalitions in this policy subsystem were identified: (1) supportive [with respect to the up-scheduling], and (2) opposing. The key evident beliefs of the supportive coalition were that the harms of codeine outweighed the benefits, and that government regulation was the best pathway for protecting consumers. The opposing coalition believed that the benefits of codeine accessible through pharmacists outweighed any harms, and consumers should manage their health without any more intervention than necessary. The policy decision reflected the influence of the supportive coalition, and this analysis highlighted the importance of their public health framing of the issue, the acceptability of their experts and supporting evidence, and the perceived legitimacy of the up-scheduling process.

**Conclusion:** Understanding these coalitions, their beliefs, and how they are translated through existing policy processes and institutions provides insight for those interested in influencing future health policy. Specific lessons include the importance of strategic frames and advocacy, and engagement with formal policy processes.

## Background

**Key Messages**

** Implications for policy makers**

- The favourable framing of policy problems and solutions to relevant decision-makers was important for the scheduling change.
- Institutional structures and feasibility need to be considered when advocating for alternative policy proposals.
- Understanding the framing and institutional structures present in this up-scheduling decision may provide learning opportunities for other similar health policy decisions, such as the supply and access of medical cannabis or nicotine e-cigarettes.

** Implications for the public**

In Australia, low-dose codeine analgesic products were classified into a more restrictive category, or ‘up-scheduled’ — from being available at pharmacies under pharmacist supervision to requiring a prescription from a doctor — due to increasing opioid-related harms. Given the potentially large impact this decision would have on consumers and healthcare professionals, and the large amount of public attention and consultation it received, we wanted to investigate how this decision was made, particularly how stakeholders’ and policymakers’ beliefs influenced policy. We found that the decision-makers believed that the harms of codeine outweighed its benefits, that medical practitioners were better placed to manage codeine supply than community pharmacists, and that government regulation via up-scheduling was appropriate to safeguard public health. Understanding this may provide learning opportunities for stakeholders and policymakers in developing other policies that regulate access to substances such as medical cannabis or nicotine e-cigarettes, with the goal of improving public health.

Internationally and in Australia, there has been increasing concern over opioid misuse and related harms.^1-4^ In response, many policies have been implemented that aim to reduce opioid-related harms, encompassing the supply and access to opioids, medication management in patients on opioid analgesic therapies, and access to treatments for opioid use disorder.^5^

 Scheduling is a legislative instrument designed to control the availability of substances to the public^6^; medicines and poisons are classified according to the level of regulatory control required to ensure public health and safety. ‘Up-scheduling’ involves placing a greater restriction on the availability of a substance, with the aim of improving therapeutic use and decreasing inappropriate use.^7^ This is especially important for substances that require intervention and monitoring from clinicians, or those that have a high risk of adverse effects or toxicity. For example, most up-scheduling research to date has focussed on the effect of the US Drug Enforcement Administrations’ decision in 2014 to up-schedule hydrocodone-containing products. Evaluation of the policy’s clinical effect has demonstrated a decrease in the number of dispensed hydrocodone prescriptions, with inconclusive effects on the prescribing patterns of other prescription opioids.^8-11^

 Codeine-containing preparations have been available in Australian community pharmacies for the treatment of pain (such as headaches, and cold and flu symptoms), and as an anti-tussive.^12^ These products were widely used, with studies showing that in 2013, 56% of the 27 million packs of codeine-containing analgesic products sold at pharmacies were provided without prescription^13^ and accounted for 37% of all opioids sold.^14^ Despite its relative low potency, the long-term use of codeine-containing analgesics has been associated with harms — overuse of combination products with paracetamol can result in hepatotoxicity; overuse of combinations with ibuprofen can cause cardiovascular, renal, and gastrointestinal adverse effects; and codeine itself as an opioid can cause dependence, tolerance, and gastrointestinal and neuropsychiatric effects.^15^

 In Australia, medicines are usually classified in Schedules 2, 3, 4, or 8, as defined in Box 1. Since 2010, codeine-containing products have been classed as Schedule 3 (Pharmacist Only Medicine), with the exception of codeine/phenylephrine cold and flu preparations, which were Schedule 2 (Pharmacy Medicine).^16^ However, in mid-2015, an external application from the Pain Management Unit at the Royal Adelaide Hospital was made to the national drug regulator, the Therapeutic Goods Administration (TGA), to up-schedule all codeine-containing products to Schedule 4 (Prescription Only Medicine).^17^ (This was only revealed after our freedom of information request to the TGA, described in further detail below). After a period of consultations and public submissions, the TGA announced on December 20, 2016 that all products containing codeine would be up-scheduled to Schedule 4 from February 1, 2018.^18^

**Box 1. DescriptionoftheSchedulesofSubstancesAvailableFromPharmaciesinAustralia**

**Schedule 2 (Pharmacy Medicine):** substances that may require advice from a pharmacist and which should be available from a pharmacy.

**Schedule 3 (Pharmacist Only Medicine):** substances that requires advice from a pharmacist but should be available without a prescription.

**Schedule 4 (Prescription Only Medicine):** substances that can only be obtained with a prescription from a prescriber.

**Schedule 8 (Controlled Drug):** substances that can only be obtained with a prescription from an authorised prescriber and have additional rules for producing, supplying, distributing, owning, and using them.

This policy decision had the potential to affect many consumers’ health, as well as the clinical practice of various healthcare professionals. In a time where the community pharmacy sector has been undergoing significant changes to their role in managing health conditions, the decision also had broader policy implications for the future role of pharmacists in consumer access to other pharmaceutical products. Understanding the policy processes and influencing factors of this decision will provide insight into similar health and pharmaceutical policy decisions to inform future policymaking. There has been no scholarly evaluation of the development of this policy; therefore, using the Advocacy Coalition Framework (ACF) for analysis, this study aims to explore the different beliefs of policy actors for this health policy issue, and to understand how these beliefs influenced the decision to up-schedule codeine.

## Methods

###  Study Design

 This study is a retrospective policy analysis, drawing on case study research methodology and using documents and semi-structured interviews as information sources.

####  The Advocacy Coalition Framework

 To guide the data collection and analysis for this study, we used the ACF, first developed by Sabatier in 1988,^19^ which has previously been used to analyse drug policy^20^ and public health policy.^21^ The ACF was relevant for this study because it allows for an analysis of policy actor participation within the defined policy process of up-scheduling.

 The ACF portrays the process of policymaking as an adversarial competition, where people engage in politics by forming advocacy coalitions who share their core beliefs, then compete with other coalitions to transform their beliefs into policy. This occurs within a policy subsystem (a particular policy area), with wider contextual factors that can influence the subsystem and provide opportunities or constraints to the coalitions. Coalitions may use research or information, public opinion, relationships with decision-makers, media, and government advisory bodies to have their views and beliefs dominate.

 Actors are bound together in coalitions because of their shared ‘belief systems,’ which encapsulate their understanding of policy problems and solutions. There are three types of beliefs: (1) *deep core beliefs*, regarding an actor’s fundamental beliefs and understanding of the world and society; (2) *policy core beliefs*, regarding an actor’s underlying beliefs on a specific policy area, including its importance, cause, and solutions; and (3) *secondary beliefs*, regarding the specific implementation of policies. The ACF suggests that deep core and policy core beliefs are the least susceptible to change; however, secondary beliefs may change, depending on circumstance, via policy-oriented learning. As core beliefs are resistant to change, advocacy coalitions within a policy subsystem may remain consistent for decades; however, external ‘shocks’ to the subsystem (such as the election of a new government) may change the nature of these coalitions.

 The scope of the ACF includes the three theoretical foci of advocacy coalitions, policy change, and policy learning.^19^ While the ACF provides a broad illustration of policy processes and these 3 foci are intertwined, it may be more feasible for analyses utilising this framework to focus on one of these foci.^22^ In our analysis, we have focused relatively more on the theoretical emphasis of policy change because we focused on a policy decision that had been made; this guided the data that were collected and analysed. For example, we first collected data to answer questions regarding key policy actors, coalitions that were formed and their beliefs, the resources and strategies used by coalitions, and any changes to policy core or secondary beliefs held by coalitions. Then, we analysed how these interacted to drive the change in codeine scheduling.

###  Data Sources

####  Documentary Data

 Documentary data provided information on the beliefs of individuals and groups, through public submissions, news reports, and media releases or position statements from representative groups. Additionally, documents detailing information about the policy process (from federal/jurisdictional parliaments, government departments, and news reports) were obtained to provide the context needed to understand actor influence.

 We began by retrieving all publicly available documents from the TGA’s codeine information hub,^23^ which included: reports outlining the interim and final decisions to up-schedule codeine-containing analgesics from Schedule 3 to Schedule 4; notices calling for public consultations; public submissions; published safety and efficacy reports,^24,25^ and economic evaluations^26^; meeting statements from TGA advisory committees; TGA media releases; and TGA training and education resources for healthcare professionals and consumers. Additionally, we submitted a freedom of information request to the TGA, in which we requested and received the initial application to up-schedule codeine-containing analgesics from Schedule 3 to Schedule 4; minutes for the two relevant scheduling committee meetings; and email correspondence between the TGA and various parties regarding requests for safety, efficacy, and economic evaluations to be performed.^17^

 Following this, we searched Factiva (a news aggregation platform) for any reference to ‘codeine’ between January 1, 2015 and February 28, 2019. This time period was chosen to capture a few months before the 2015 application to up-schedule codeine, to a year after the implementation date. This search retrieved news reports and transcripts from radio and television interviews, excluding duplicate and identical records. From this Factiva search, a total of 153 records were retrieved.

 As this policy decision was widely discussed, debated, and politicised, we also searched the Australian Hansard, which includes reports of proceedings of the Australian parliament and its committees. Similar to the Factiva search, we included any documents with reference to ‘codeine’ between January 1, 2015 and February 28, 2019. Additionally, we attempted to search publicly available State/Territory ministerial diaries from January 2015 to March 2019 inclusive, to identify when and which interest groups were meeting with jurisdictional Ministers for Health about codeine and pain management matters. This data was only available for the New South Wales Minister for Health and Minister for Medical Research.^27^

 After examining these sources, we were able to identify the key stakeholder groups involved in this decision, and subsequently searched their websites for media releases and position statements on this codeine up-scheduling decision. Further key documents were also iteratively identified through interviews with participants. Altogether, we analysed documents from the TGA (236 public submissions, 4 published reports or reviews, 13 documents obtained via the freedom of information request, 2 advisory meeting statements, and 3 decision documents); 153 news reports from Factiva; 3 Hansard transcripts; and 7 position statements, media releases, or reports published by peak bodies. It is important to note that many peak bodies’ statements were contained within the submissions to the TGA.

####  Semi-structured Interviews

 While documentary data provided stated or public interests, semi-structured interviews provided further insights, opinions, and explanations regarding influences on the policy process. Between February 2020 and March 2021, the lead author conducted 14 semi-structured interviews with 15 key informants, identified through purposive sampling based on the findings of the documentary data. Interviews lasted 40-63 minutes (average 52 minutes), and additional potential interviewees were identified through snowball sampling. Participants identified as members of healthcare professional representative bodies (n = 7); staff of government health departments (n = 3); practising clinicians (n = 8); researchers and academics (n = 3); consumer representatives (n = 1); and representatives from the manufacturing/wholesaler sector (n = 3). Eight participants identified as having multiple roles. We also sent interview requests to nine other individuals; however, we received no response from eight individuals and one request was declined.

 An interview guide was developed, informed by the study framework. It covered questions on participants’ opinions about codeine up-scheduling; their experiences with the policy development; their view on influential policy actors; and their view on the role of evidence in the process. The interview guide was piloted in a qualitative analysis research group prior to the first interview.

###  Data Analysis

 The data from documentation and interviews were triangulated to corroborate findings for a more accurate understanding of the policy process.

 Qualitative content analysis was performed on collected documents. Interviews were audio recorded (where permitted by the participant) and transcribed. All documents, available interview transcripts, and field notes were imported into the qualitative data analysis software, NVivo 12 Plus (QSR International).

 Coding was performed both deductively according to the analytical framework, and inductively. To understand the up-scheduling process, we used the policy cycle heuristic (which suggests policy proposals move through stages of agenda-setting, policy formulation, decision-making, implementation, evaluation, and maintenance, succession, or termination)^28^ to map the sequence of key events and construct a chronology. Additionally, we coded the institutional, political, and sociocultural norms and context relevant to this decision. To understand the coalitions’ beliefs, we coded the key policy actors involved; how they defined the problems and solutions in the policy subsystem; and the underlying beliefs that were characteristic of the coalitions. The results are structured around the broad themes that were constructed; these centred around the role of pharmacists and healthcare professionals, perceptions of codeine, impact on consumers, and the role of government in medicines regulation.

 All data was coded by one author (KC), who kept a reflexive journal during data collection and analysis. Additionally, co-authors LB and AMT, and a qualitative analysis research group were consulted at regular intervals during data collection and analysis to ensure validity in findings.

###  Research Rigour and Reflexivity

 The study design was also informed by the positionality of the researchers. KC is a doctoral student receiving training to quantitative and qualitative research methods, and is also an early-career pharmacist practising in community pharmacy. LB and AMT have used mixed methods approaches to study health policy development globally.

## Results

###  Narrative Chronology

 On February 1, 2018, the TGA up-scheduled codeine-containing products^29^ from Schedule 2 and Schedule 3 to Schedule 4. This followed three years of dialogue and interaction between the decision-makers (the TGA) and various stakeholders.

 This process began in February 2015, when the Director of the Pain Management Unit of the Royal Adelaide Hospital submitted an application to the Medicines and Poisoning Scheduling Secretariat for the re-scheduling of codeine-containing medications from Schedule 3 to Schedule 4. The application referenced a previous scheduling decision made in 2010 to up-schedule codeine-containing products from Schedule 2 to Schedule 3, and proposed that these products should be further up-scheduled in line with factors required for Schedule 4 due to its potential for misuse and dependency, and severity of adverse effects.

 In response, the medicines scheduling delegate in April 2015 published a pre-meeting public notice, detailing the scheduling proposal for consideration by the Advisory Committee on Medicines Scheduling (ACMS) at the July/August 2015 meeting, with the main item:

 “*to delete the Schedule 3 entry for codeine, and re-schedule the current schedule 3 codeine entry to Schedule 4 due to potential issues of morbidity, toxicity and dependence …” *

 This decision was consultative and data-driven; in making the decision, the TGA drew on the ACMS’ advice, invited public consultation, and requested data concerning codeine’s safety and efficacy, and the economic impacts of up-scheduling. Notably, there were three rounds of public consultation before a final decision was made — a total of 236 submissions were received, ranging from individual consumers; individual practising clinicians; peak healthcare professional bodies; pharmaceutical industry and manufacturers; and consumer organisations. The majority of these submissions were against the up-scheduling, driven by individual consumers who used Schedule 3 codeine analgesics.

 Despite opposition through submissions and direct lobbying, in December 2016, the TGA confirmed its interim decision to up-schedule codeine, based on its relatively low safety and efficacy profile. Given this decision had the potential to affect a large number of consumers, patients, and healthcare professionals, they conducted various communication and implementation activities to assist in the transition. A Nationally Coordinated Codeine Implementation Working Group (NCCIWG) was established to assist with implementing communication and engagement strategies. Additionally, the TGA organised a series of workshops around Australia for healthcare professionals; these were led by clinical experts and communicated the changes to codeine access, and provided management options to help individuals with acute/chronic pain.

 At the same time, within this window between the decision and implementation, opposition to the up-scheduling continued. The Guild and the Pharmaceutical Society of Australia met on multiple occasions with federal and jurisdictional Health Ministers and Members of Parliament, and proposed alternative solutions to up-scheduling. However, despite these lobbying efforts, codeine-containing products were up-scheduled to Schedule 4 on February 1, 2018.

###  Coalitions and Their Framing of Problems and Solutions

 This analysis identified two main coalitions identified in this policy subsystem: (1) supportive [of the up-scheduling]; and (2) opposing. Both coalitions acknowledged that there were harms and benefits to having codeine analgesics available under pharmacist supervision. However, they differed in their conceptualisation of harms and benefits regarding the role of healthcare professionals; codeine as a substance; consumer experience of codeine use; and the role of the government in protecting consumers from harms (summarised in Table).

**Table:** Coalitions’ Framing of the Benefits and Harms of Codeine Up-Scheduling

	**Role of Healthcare Professionals**	**Codeine as a Substance**	**Consumer Experience of Codeine Use**	**Role of the Government**
Coalition 1 — supportive	Pharmacists and pharmacies are inadequate gatekeepers of codeine as a Schedule 3 substance the harms are not mitigated by pharmacists and pharmacists are upselling its benefits	Codeine is inherently a terrible drug as it is not effective and there is high risk of adverse events that harms outweigh benefits are inherent to the properties of the drug Codeine at Schedule 3 low doses do not meet the Schedule 3 criteria in the Scheduling Policy Framework codeine does not fit the harm/benefit profile for Schedule 3 substances	Consumers do not necessarily understand codeine as an opioid or what it should be used for misunderstanding or wrong perception of their relative harms and benefits There are many individuals who have become addicted to codeine, and there have been increases in deaths and illness from codeine use harms are very serious	The government and drug regulator has a role in safeguarding the public has a role in signalling that harms outweigh benefits
Coalition 2 — opposing	Pharmacists are sufficiently trained and good custodians of codeine while there are harms associated with codeine use, pharmacists are best placed to identify them and refer them to GP the harms arise from a lack of pharmacist supervision	It is not Schedule 3 low-dose codeine that is the problem, it is the other substances that are used concurrently or will be used instead of codeine that are the problem the benefits of codeine as pain relief still hold as the harms are from other substances	The majority of consumers are “legitimate users” and should not be “punished” for the minority who experience harms or are “abusers” ‘I benefit from codeine; harms are what other people experience’ Pain has been left undertreated and patients will be left to suffer the harm is not having pain relief	The government is overstepping its role and up-scheduling is the action of an “overbearing nanny state” the government does not and would not know the benefits of codeine for individual consumers and should not ban it because a minority are affected by the harms

Abbreviation: GP, general practitioner.

####  Coalition 1: Supportive

 The coalition supportive of the decision to up-schedule codeine-containing products included general practitioners (GPs); addiction medicine specialists; peak bodies representing these medical practitioners; hospital pharmacists; some community pharmacists (particularly those earlier in their career or not managing a pharmacy); some consumer groups; and consumers who had had experience with codeine-related harms and misuse. It is important to note that the consumer and patient advocacy groups Consumers Health Forum and Painaustralia were initially against the up-scheduling due to the analgesic benefits reported by consumers; however, they later publicly confirmed their support for up-scheduling and became part of this first coalition after discussions with pain management specialists, and after recognising codeine’s ineffectiveness for chronic pain and the harms from its addictive properties.

 The core belief that characterised this coalition was that harms outweigh benefits, and that regulation of codeine via scheduling is the best way to protect consumers.

 This was demonstrated through their view of the role of community pharmacists — that pharmacists were inadequate gatekeepers of codeine as it existed as Schedule 3 substances. Public media statements, submissions, and interviews indicated that members of this coalition believed that the harms arising from codeine use were not mitigated by pharmacists. They also observed that in reality, there was little genuine consultation by pharmacists with consumers before a codeine product was supplied. They also suggested that pharmacists may find it difficult to refuse supply — whether because of financial interests for pharmacists working in community pharmacies, a lack of confidence to refuse supply, or pharmacy staff not reliably identifying individuals misusing codeine.

 “*We were not convinced that pharmacists were doing enough to persuade people to buy other products… we felt that basically if somebody walked in and asked for a codeine product as an OTC [over-the-counter] — they pretty much got it if they asked for it… our interpretation of a Schedule 3 is you should front up to the pharmacy, say what your symptoms are, and then the pharmacist initiates the discussion about a particular medicine… I think there was growing evidence that those conversations were just not happening in the right way” *[Consumer representative, interview].

 Members of the supportive coalition believed it would be better for medical practitioners to manage and be responsible for the use of codeine as an analgesic, and for individuals requiring pain relief (especially for chronic pain) to see a doctor for a “proper” diagnosis and prescription. They argued that if codeine were kept at the Pharmacist Only level of access, there would be a bigger risk of dose escalation without appropriate clinical supervision.

 Some addiction medicine specialists acknowledged that while up-scheduling codeine to Prescription Only would not remove the risks from codeine use and GPs may also prescribe it inappropriately, it was still preferable to having it available in community pharmacies and supplied at the discretion of pharmacists.

 This coalition also considered codeine as inherently a problematic drug, viewing it as ineffective with high risks of adverse events. Research evidence (trials and reviews) were quoted to emphasise codeine’s relative ineffectiveness at the low-doses available in Schedule 3 products. Crucially, they argued that codeine does not add enough benefit to warrant the risks, and if it does not provide sufficient analgesia, there is a risk and tendency for consumers to take excess, leading to additional harms from overuse of paracetamol or ibuprofen in codeine combination products.

 Another key argument against codeine itself was the significant genetic variability between individuals that determines how well codeine works as an analgesic; this variability in response and lack of feasible genetic testing suggests that codeine is not a clinically useful or good medicine.

 The supportive coalition was also concerned about consumers’ lack of understanding of codeine as an opioid, and that consumers had a misunderstanding or wrong perception of the relative harms and benefits; this supported the coalition’s core belief that consumers needed to be protected and why regulation was necessary. Healthcare professionals in support of up-scheduling noted in their experience that ‘opioids’ were being referred to as strong prescriptions or heroin (rather than including substances like codeine), and that codeine had been misrepresented as a vital, strong analgesic. In addition, they had observed individuals using codeine for chronic pain or sleeping difficulties, suggesting that they did not know what it should be used for, or that their use should have been under guidance from medical practitioners, not pharmacists. Importantly, a few of the interviewed participants identifying with this coalition had also seen first-hand the seriousness of harms from codeine use, particularly, subsequent illnesses, addiction, and deaths. This reinforced their belief that up-scheduling was necessary to protect consumers and the public.

 “*A lot of consumers don’t understand that codeine is an opioid. Opioids in people’s minds are heroin and perhaps morphine… they didn’t see codeine as an opioid, and I think there wasn’t an appreciation of the harm that codeine can do”* [Consumer representative, interview].

 Lastly, supporters of up-scheduling saw the government and drug regulator as having a broader role in safeguarding the public when harms outweigh benefits. When interviewed, some members in this coalition praised the conduct and integrity of the TGA, regarded them as a trusted regulator, and believed their authority should not be undermined. Some participants considered how the TGA regulates access to medicines as a signal for clinical practice, and that keeping codeine as Schedule 3 undermines messaging to encourage consumers to look at other pain management options, not just pharmacotherapy.

 “*Our current arrangements are not serving the public good, by perpetuating last-century prescribing habits and providing drugs freely which are neither [particularly] safe or particularly effective” *[Pain specialist, news article].

 One interview participant pondered the broader social and policymaking ramifications of not up-scheduling codeine — if an enlightened society is “built on a bedrock of good science and critical thinking” and science shows that codeine is ineffective and harmful, then what does it mean for society if policymakers let codeine stay at this level of accessibility despite evidence that it should not be?

####  Coalition 2: Opposing

 The coalition against the decision to up-schedule codeine included peak bodies representing community pharmacy owners and pharmacists; some consumer groups; and consumers who used codeine analgesics. The consumer and patient advocacy groups Consumers Health Forum and Painaustralia were initially in this coalition; however, became publicly supportive of up-scheduling after reconsidering the evidence of codeine in its current scheduling as more harmful than beneficial.

 The core belief that characterised this coalition was that benefits outweigh harms, and that consumers and patients should be able to manage their health and know what is best for themselves with no more intervention than is necessary.

 In contrast to the first coalition, those opposed to up-scheduling had a different view of pharmacists’ roles, arguing that pharmacists are sufficiently trained and good custodians of codeine. Given that many community pharmacists were part of this coalition, they maintained that pharmacists are more than capable of making clinical decisions for the appropriate supply of codeine, and to suggest otherwise would be a “professional slap to the face.” To up-schedule would also place a limit on the professional services provided by pharmacists; if patients needed relief for acute pain, pharmacists would have a “decimated toolbox.”

 Pharmacists and consumers in this coalition also argued that pharmacists were best placed to identify patients who experience harms, should they occur. They acknowledged that while there are patients who have issues with dependence to low-dose codeine, they would be “slipping through the system” were codeine up-scheduled, due to the lack of real-time monitoring in some jurisdictions, and nationally. This was a major reason for the Guild to develop and implement their national MedsASSIST program — their solution was a real-time monitoring program specifically created to monitor Schedule 3 codeine analgesics and help pharmacists make clinical decisions for supply and referral. In many public submissions, consumers and pharmacists suggested that up-scheduling (particularly without any monitoring system) would result in people obtaining “stronger” prescriptions from their doctor or that there would be increased instances of ‘doctor-shopping,’ both of which would lead to more harms than the current situation.

 Another prominent argument from this coalition concerned the potential impacts on the health system, that not having codeine readily available would cause pressure on existing services for patients in need of adequate pain relief. It would be harder for patients, particularly in rural areas, to obtain acute relief, claiming that GP clinics, emergency departments, and after-hours doctor services would be “inundated.” They also argued that there were not enough feasible alternatives, with inadequate pain management services, drug and alcohol services, and physiotherapy being prohibitively expensive. Ultimately, they believed that there would be an avoidable system-wide large cost to the public Medicare system.

 “*If we can keep patients in the community, getting advice in pharmacy, getting treatments that actually are efficacious, that keeps the cost out of the core system. Theoretically, if all those people had pain are now visiting a doctor and charging Medicare, there’s a much higher cost to the system” *[manufacturing/wholesaler sector, interview].

 After the decision had been made to up-schedule codeine, pharmacy leaders in the opposing coalition proposed a compromise, informally coined ‘Prescription Except,’ where pharmacists would be able to supply a small quantity of codeine-containing products for individuals with acute pain. This was aligned with their view that consumers should be able to obtain some analgesia in situations where a doctor could not be accessed (eg, after hours) but did not warrant visiting an emergency department.

 Contrasting with the supportive coalition, this coalition viewed low-dose codeine as a ‘good drug’ — their views on codeine centred on its relative safety compared to other substances, suggesting that the harms observed were due to the other drugs in combination products (paracetamol, ibuprofen), or from what would be substituted if codeine were less accessible (stronger prescription opioids, illicit opioids, alcohol and other drugs). A few members of this coalition had read the evidence on harm from Schedules 2/3 codeine, and thought that the data had been misinterpreted by decision-makers, and that adverse events were not as prevalent as presented. For example, they suggested that the mortality data that was used included both over-the-counter and prescription codeine, rendering it difficult to attribute it to over-the-counter codeine only.

 The belief that the benefits of codeine far outweighed harms was seen clearly in their view that the majority of consumers benefited from codeine as “legitimate users” who should not be “punished” for the minority who experience harms or are “abusers.” Public submissions revealed that many consumers held the position that “there will always be people who are addicted to something” and framed it from an individualistic perspective — that they used it appropriately and it was other people who did not.

 Pharmacists in this coalition also noted that there was no data on the ‘successful use’ of codeine — individuals who used it appropriately and obtained effective analgesia — but that it should have been considered as evidence. From their perspective, the best strategy would be to “educate people and monitor.”

 Finally, this coalition viewed the government and the TGA as overstepping its role and that up-scheduling was the action of an “overbearing nanny state.” In interviews and many submissions, they argued that the government and health bureaucrats would not know how individuals benefited from codeine, that it was not their role to ban codeine because a minority were affected by harms, and they “can’t try to prevent people from themselves for everything.” Members of this coalition had very strong opinions on this aspect, claiming that the TGA decision-makers had a political agenda, and that this decision was an infringement on individual rights.

###  A Shift in Dominant Coalitions

 Prior to the up-scheduling, the dominant coalition was the one opposed to up-scheduling to Prescription Only, in that codeine remained available in pharmacies as Schedule 2 or 3 products. However, the application to up-schedule codeine triggered the TGA re-scheduling process, which ultimately led to a shift in dominant coalition where the supportive coalition ‘won’ within the policy subsystem, resulting in policy change aligned with their belief that the harms of codeine outweigh the benefits.

 The supportive coalition framed and defined the problem as a public health issue requiring a government regulatory response to protect consumers and patients from harms, appealing to the TGA’s regulatory role and nature. While they were able to demonstrate instances of documented harm in Australia, this policy process to up-schedule was also taking place amidst the broader context of the opioid crisis in Australia and internationally. Opioid-related misuse and harms have been given prominent attention within the medical and health community, as well as in the public. While the discourse affected individuals’ assumptions about opioids and people who use or misuse them, it also made clear the adverse health and social effects of opioid misuse, and the wide range of strategies urgently needed and developed to address this public health issue. Regulatory measures to reduce opioid supply and access have been used extensively in North America, and this context could have made a scheduling approach for codeine more acceptable. From news reports in January 2018, Australia’s Chief Medical Officer said:

 “*The decision to make codeine-containing medicines prescription from February 1 is in line with 26 other countries which have done the same thing based on good scientific evidence.”*

 The shift in dominant coalitions was also enabled by the perceived legitimacy of the up-scheduling process; it brought the public along and provided avenues for experts and evidence to be used. The TGA took an approach that involved extensive consultation, was largely transparent, and considered the impact on the public. There were three rounds of public submissions, with each round considered in the ACMS meetings, and decisions were deferred to take submissions into consideration. Even some individuals from the opposing coalition who thought the “blanket ban” was too reactionary, acknowledged that it was a robust process due to the multiple opportunities for individuals and organisations to provide input. Submissions and commissioned reports were also published during the process, improving the process’ transparency. The TGA and federal government invested heavily in implementing and communicating the up-scheduling; for example, federal funding of over $1 million was given to professional peak bodies to help inform patients and manage the transition.

 Additionally, the proximity of clinical experts (who tended to be in the supporting coalition) to the decision-makers may also have influenced the decision to up-schedule. The TGA drew on the readily available expertise of many addiction medicine and pain management specialists via their roles in expert advisory committees. They also had the resources to commission evidence conducted by experts in the form of reviews looking at the safety and efficacy of codeine, and economic impacts of up-scheduling, all of which supported the decision.

 In efforts to overturn the decision, the opposing coalition participated in significant levels of lobbying, and was seen as trying to circumvent due process. Led by the Guild, they lobbied jurisdictional and federal Members of Parliament, jurisdictional Health Ministers, and persuaded them to lobby the federal Health Minister. Members of the supportive coalition viewed the lobbying as “irresponsible and unprincipled” and reinforced their support of the TGA’s decision. Ultimately, it is unclear whether the lobbying efforts would have been effective as the federal Health Minister had repeatedly insisted the decision was made independently by the TGA — it was framed as an evidence-based, consultative, and non-political decision.

 Lastly, the main alternatives proposed by the opposing coalition were not seen as feasible. Led by the pharmacy peak bodies, they included (1) national real-time monitoring of codeine; and (2) creating an exemption for pharmacists to supply a small quantity of codeine for acute pain, in conjunction with a monitoring program. However, neither of these solutions were successful due to the existing institutional structures relevant to an up-scheduling decision. These decisions are the remit of the TGA, but they do not have the mandate to develop jurisdictional or national real-time monitoring programs. Currently, these programs are developed and implemented independently in each jurisdiction — some jurisdictions do not have a program or infrastructure in place, nor has a national program been developed. Similarly, a ‘Prescription Except’ compromise would require each jurisdiction to have an operational monitoring program, and each jurisdiction’s poisons legislation to be amended. As was evident in the up-scheduling decision, while jurisdictions are in charge of their own regulation, they prefer to be consistent with other jurisdictions. In this situation, the Guild led the proposed solution, but it appeared they were unable to persuade any and all jurisdictions to adopt the measure.

## Discussion

 In this policy process, we identified two main coalitions characterised by different beliefs on the harms and benefits of codeine up-scheduling, who sought to influence the policy decision. This decision was not just a debate about the balance of risks and benefits of codeine; it also concerned the risks and benefits of government intervention, and the risks and benefits of codeine access via different health professionals — these other aspects affected the overall harm-benefit ratio.

 While this up-scheduling occurred in Australia within a specific policy architecture and context (the role and transparency of the regulator, the existence of a ‘bridging’ schedule between Prescription and Pharmacy Only, and federal/jurisdictional legislation enactment) regulation of similar substances is universal. Regulators need to consider harms and benefits while navigating a variety of interests on regulating these substances, and this has also been seen in decisions around medical cannabis and nicotine e-cigarettes.

 In our analysis of codeine up-scheduling, we identified that the framing of the policy problem (driven by underlying beliefs) was important for advocating for policy change. The successful supportive coalition framed the codeine up-scheduling as a necessary tool to address the public health issue of increasing opioid use and harms, where the risks of codeine outweighed benefits, and where regulation was necessary to protect consumers. Similar to codeine, there are different beliefs regarding the effectiveness of cannabinoids for treating neuropsychiatric conditions that have limited pharmacotherapeutic options, its abuse potential, and the sociocultural implications for increased access.^30^ Likewise, nicotine e-cigarettes have been suggested as a means for smoking cessation^31^; there is growing debate in countries like the United Kingdom,^32^ the United States,^33,34^ and Australia^35^ over their safety profile, effectiveness as smoking cessation tools, and potential as a gateway to other nicotine delivery systems.^36-38^

 Analysing the codeine up-scheduling decision allowed us to examine how a successful coalition could present risk/benefit beliefs — regarding the substance’s safety and effectiveness, and the role of health professionals and government in managing its access — and demonstrated the importance of framing in policy advocacy. In other drug policy decisions (cannabinoids and e-cigarettes) that may have the same policy actors and types of beliefs, our analysis may provide insights into teasing out potentially similar frames embedded into debates, and how actors can interact with similar institutional processes to facilitate translating beliefs to policy action.

 The use of the ACF also highlighted instances of policy learning, where two key consumer groups (Consumers Health Forum and Painaustralia) changed coalitions to eventually support the up-scheduling decision. Faced with what they perceived to be strong evidence of the harms of leaving codeine in its current schedule, these groups re-evaluated their position and found that up-scheduling would better align with their priorities. Policy champions was another element of the ACF evident in this case study, where the opposing coalition appeared to be led by the groups representing community pharmacy interests, and the supporting coalition by medical practitioners, including specialists. Policy brokers are generally actors that aim to minimise conflict and foster compromises between coalitions; this was most clear in the implementation stage, with the TGA-facilitated multidisciplinary implementation group (NCCIWG).

 While community pharmacy owners and managers may have objected to the up-scheduling because of the potential loss of revenue and business, this analysis revealed that their concern was more on the impact up-scheduling would have on their professional identity — it would reduce their scope of practice and the extent to which they could clinically help patients. This up-scheduling decision occurred during a time when there has been much discussion and change around expanding pharmacists’ scope of practice,^39,40^ with pharmacist prescribing^41,42^ at the forefront of this agenda. This case study demonstrated how re-scheduling decisions may be a proxy discussion for which healthcare professionals are best suited to manage a substance’s supply and access, and raises deeper questions about pharmacists’ professional capacity and identity, particularly with regards to prescribing.

 Schedule 3 effectively functions as a means for pharmacists to prescribe, and any decisions (like codeine up-scheduling) where products are re-scheduled to and from this Schedule will affect community pharmacists and GPs. In Australia, recent discussions have centred around the use of Appendix M in the national *Standard for Uniform Scheduling of Medicines and Poisons.*^43^ This is a new category that could facilitate the down-scheduling of Schedule 4 substances to Schedule 3, with additional controls to ensure appropriate use. Pharmacy peak bodies and community pharmacy advocates have been supportive of increased accessibility of medicines to consumers and patients via pharmacists, and view Appendix M as a possible pathway to achieve this. It would also recognise pharmacists’ clinical skills and knowledge, and provide them with more responsibilities and opportunities to practice independently.^44^ As of July 2021, no medicines have been added to Appendix M, and this is an area in which more discussions between pharmacy advocates and the TGA may occur, with the potential to impact public health.

 Pharmacists in other contexts have also been vocal about changes to medicines policy in the area of pharmacist prescribing. A variety of prescribing models have been implemented across countries like Canada,^45^ the United Kingdom,^46^ and the United States.^47^ These differ with level of pharmacist autonomy, clinical situations, and the medicines that pharmacists are permitted to prescribe,^41^ and pharmacists are continually advocating for a larger prescribing scope to reflect their expertise.^48,49^ Our up-scheduling analysis illustrates that, regardless of context, persuasive framing of professional capacity and identity is necessary for the pharmacy sector to advocate for these increased prescribing opportunities, and this finding contributes to the literature on pharmacy policymaking.

###  Limitations

 Interviews for this study were conducted between February 2020 and March 2021, coinciding with the coronavirus disease 2019 (COVID-19) pandemic, and it is possible the health crisis may have affected other individuals’ willingness to participate. However, this research also drew on substantial documentary data, and triangulating the data from documents and interviews demonstrated that a comprehensive account of the policy process could be obtained.

 Another difficulty with recruitment was the lack of retrospectively publicly available information regarding the members of the ACMS and other key decision-makers within the TGA and jurisdictional health departments. Even after extensive document searching and asking interview participants (who could not recall, or did not know them), we could not confirm and identify these individuals, and therefore, could not contact them for recruitment. Although we could not interview these key actors, we were still able to understand the mechanics of the policy process, analyse the TGA’s perspective through published documents outlining their decisions and arguments, and obtain some relevant perspectives about the process from non-TGA personnel.

 Although some interview participants had experience working in regional or rural communities, most interview participants were primarily working within metropolitan areas and state capital cities. This may have affected beliefs and perceptions, particularly around issues of access to codeine and other health services. However, both coalitions had participants who were able to discuss their experiences in these communities and how the up-scheduling would impact consumers and health professionals. Additionally, many public submissions were submitted by individuals and groups from regional and rural Australia, and their views were captured as part of the document analysis component.

 Given the retrospective nature of this study, it is also possible that interview participants’ accounts of the process have changed and been affected by external events in the years since the policy development and implementation, instead providing an account that retroactively justifies decisions that were made. While this may affect our understanding of the ‘true’ account of the policy development, this was not ultimately the goal of this study. Instead, we sought to examine the beliefs of the policy coalitions and how they developed into policy action; these beliefs and perceptions of the policy problems and solutions were still clearly evident at the time of this analysis.

## Conclusion

 This analysis identified two advocacy coalitions (supportive and opposing codeine up-scheduling) with different views on balancing risks and benefits of codeine use. The successful supportive coalition believed that the harms of codeine accessible as Pharmacist Only Medicines outweighed the benefits, and were able to frame their beliefs and use the institutional structures to achieve their desired policy outcome. Although Australia’s regulatory architecture for drug policy decisions may be unique, an understanding of actor beliefs and participation in this codeine up-scheduling can provide insight into other health policy areas in other contexts.

## Ethical issues

 Ethics approval was obtained from the University of Sydney Human Research Ethics Committee (Project No. 2019/863).

## Competing interests

 Authors declare that they have no competing interests.

## Authors’ contributions

 Conception and design: KC, AMT, and LB. Acquisition of data: KC. Analysis and interpretation of data: KC, AMT, and LB. Drafting of the manuscript: KC. Obtaining funding: PhD scholarship obtained by KC. Supervision: AMT, LB.

## Funding

 KC is supported by The University of Sydney’s PhD Scholarship in the Community Pharmacy — Research into Policy.
